# International Travel and Sexually Transmitted Disease

**DOI:** 10.3201/eid0912.030210

**Published:** 2003-12

**Authors:** Paul Etkind, Sylvie Ratelle, Harvey George

**Affiliations:** * Massachusetts Department of Public Health, Jamaica Plain, Massachusetts, USA

**Keywords:** sexually transmitted disease, travel medicine

**To the Editor**: Recent articles in the professional literature (*1*–*3*) have offered advice regarding the importance of taking a careful travel history, particularly in this time of unprecedented levels of international travel (*4*). Such screening serves an important public health purpose as well, especially for sexually transmitted disease (STD) control.

Sexual behaviors associated with travel can change the level of risks for STD transmission (*5*–*7*), and the epidemiology of STDs is not uniform throughout the world (*8*,*9*). These geographic differences may increase the risk of a traveler’s becoming infected, or, conversely, increase the risk of a traveler’s introducing a sexually transmitted pathogen, possibly one that is resistant to treatment, into a low-incidence area (*10*). In addition, different strains of pathogens may be common in different parts of the world (*11*–*14*). For example, quinolone-resistant *Neisseria gonorrhoeae* (QRNG) is much more common in Asia (up to 40% of all isolates) (*15*). These strains of QRNG were first introduced in the United States by persons who engaged in sexual activity abroad, but now California and Hawaii have an increasing incidence of infection attributable these strains (*16*). Indeed, QRNG has become endemic in those states, and incidence is no longer related to travel. During 1999–2001, only 3 QRNG isolates (0.28%) were identified among the 1,066 synococcal isolates cultured in the STD Laboratory, State Laboratory Institute, Massachusetts Department of Public Health (Massachusetts Department of Public Health, unpub. data). However, in 2002, 9 (2.1%) of 425 isolates of *Neisseria gonorrhoeae* were quinolone resistant. None of the persons recently infected reported a history of travel outside of New England. Unfortunately, few had reliable information to identify their partner(s). Those partners who were identified were either not located or did not agree to speak with the disease intervention specialist.

This experience with antimicrobial resistance of *Neisseria gonorrhoeae* should serve as a model for STD prevention planning and programming. It highlights the importance of retaining the laboratory capacity to monitor antimicrobial susceptibilities of bacterial STD isolates. Treatment protocols should be adjusted in light of the prevalence of resistant strains of sexually transmitted pathogens. In cases in which symptoms associated with a bacterial STD persist after what is usually considered appropriate treatment, clinicians should obtain cultures and perform susceptibility tests on isolates. Nucleic acid amplification technologies do not provide critical antibiotic susceptibility information. In this situation, the public health STD program or laboratory should be contacted for guidance. Determining the sensitivity pattern of the pathogen in an expeditious fashion will ensure that appropriate and timely therapy can be initiated for the infected patient as well as enable more effective follow-up and treatment to sexual contacts. Asking patients who seek treatment for a possible STD about their own and their partner’s travel histories is important to broaden the differential diagnosis (*17*). The increase in population mixing facilitated by travel and Internet-generated contacts may be diminishing the importance of the focality of traditional STD epidemiology. Finally, STD prevention messages should be a part of the health advice offered to travelers (*7*,*18*,*19*).
